# Supplementary material for: Evolutionary basis of intermale sexual behavior by multiple pheromone switches in Drosophila
Source: bioRxiv. 2025 Nov 14:2025.10.14.682417. Preprint. [Version 2] doi: 10.1101/2025.10.14.682417 (PMC12632756; doi:10.1101/2025.10.14.682417)

bioRxiv preprint doi: https://doi.org/10.1101/2025.10.14.682417; this version posted November 14, 2025. The copyright holder for this preprint (which was not certified by peer review) is the author/funder, who has granted bioRxiv a license to display the preprint in perpetuity. It is made available under aCC-BY 4.0 International license.

Supplemental Figure Legends

Figure S1. Additional behavior ethograms and locomotor analyses of *Drosophila* intermale social interactions

(A) Ethograms from twenty-minute interactions depicting aggressive and courtship actions scored in three representative conspecific male-male pairs per species across various *Sophophora* and *Drosophila*. Nodes are bout counts of a given action type, with sizes representing frequency normalized to summed bouts scored across all actions (expert annotation). Edges indicate action transitions, with weights representing the fraction of bouts of a given action (arrow origin) for which a second action (arrow destination) occurred within one second. Nine total actions in each ethogram are arranged in five rows: first, two aggressive threat actions (threat, pump) (*106*); second, two aggressive contact-mediated actions (lunge, hold) (*49*); third, two additional contact-mediated aggressive actions (headbutt, barrage) (*157*); fourth, two courtship actions utilizing the wings and typically directed toward females (UWE, BWE) (*20*); fifth, copulation attempt. Nodes indicating aggressive actions are filled with blue shades and courtship actions with reds, except for headbutt (white) and barrage (black). Species arrangement and divergence times according to compilation of available phylogenies (*75*, *154*, *155*). Gray coloration indicates that very few social actions of any kind were observed. Abbreviations: UWE, unilateral wing extension; BWE, bilateral wing extension; Cop. att., copulation attempt. Barrage described previously in *D. pseudoobscura* (*136*) but named here.

(B-E) Locomotor summaries for all fifteen species included in the behavioral screen. Per-frame calculations of each fly's forward velocity (B), angular velocity (C), facing angle relative to the partner (D), and inter-fly distance (E) are derived from automated tracking of both flies (*56*) in all recorded male-male pairs. Distributions shown as violin plots with dotted means. *D. santomea,* orange; *D. yakuba*, green.

bioRxiv preprint doi: https://doi.org/10.1101/2025.10.14.682417; this version posted November 14, 2025. The copyright holder for this preprint (which was not certified by peer review) is the author/funder, who has granted bioRxiv a license to display the preprint in perpetuity. It is made available under aCC-BY 4.0 International license.

Figure S2. Performance and usage of automated behavior classifiers for intermale social interactions within the *melanogaster* subgroup and additional *D. santomea* strains

(A,B) Automated behavior classifier performance for lunge (A) and unilateral wing extension (B). Three twenty-minute representative recordings of male-male pairs for each species were manually scored for both actions (expert annotation, same pairs as in Fig. 1A) and exposed to automated behavior classifiers trained previously for *D. melanogaster* (*57*), implemented in JAABA (*55*). Manual (x-axis) vs. classifier-generated annotations (y-axis) are compared in $\log_{10}$ scale, using either the number of bouts (lunge) or their cumulative time duration (UWE). Solid lines, diagonals; dashed lines, linear fits. Adjusted R^2, significance by F-tests for fits, and Pearson correlations (r) indicated at bottom right. *D. melanogaster*, black; *D. santomea,* orange; *D. yakuba*, green; *D. simulans*, *D. mauritiana*, *D. sechellia*, *D. erecta*, gray. Note near-perfect overlap with the diagonal for lunge and strong correlation for UWE despite classifier false positives in cases with few manual annotations (all subgroup species except *D. santomea* show very little male-male courtship).

(C,D) Aggression (lunge, C) and courtship (UWE, D) measurements from automated classifiers in all recorded male-male pairs for *melanogaster* subgroup species and additional *D. santomea* strains. *D. santomea* 00 (also called STO.4) is the strain included in the behavioral screen and used throughout as "wildtype" (National Drosophila Species Stock Center 14021.0271.00). Strains 146 (STO-CAGO 1482), 148 (STO.7), and 151 (STO.6) also derive from São Tomé from females collected between 1100 and 1500 m elevation (*117*). Boxplots show full distribution range within whiskers (excluding statistically identified outliers) and second and third quartiles within boxes, with medians in bold. Individual data points (including outliers) overlayed as gray dots. Outliers retained in all summary metrics and statistical comparisons here and throughout. Lettered statistical groupings assigned by post-hoc Dunn's tests following significant Kruskal-Wallis. Note low aggression and high courtship conserved in *D. santomea* strains (orange), low aggression and courtship in the sibling species *D. yakuba* (green), and high aggression and low courtship in *D. melanogaster* (black).

(E,F) Summaries of penetrance (x-axes) and expressivity (y-axes) for aggression (E) and courtship (F) across subgroup species and *D. santomea* strains. Metrics derived from data in (C,D).

bioRxiv preprint doi: https://doi.org/10.1101/2025.10.14.682417; this version posted November 14, 2025. The copyright holder for this preprint (which was not certified by peer review) is the author/funder, who has granted bioRxiv a license to display the preprint in perpetuity. It is made available under aCC-BY 4.0 International license.

Figure S3. Intermale aggression and courtship in *melanogaster* subgroup species with fruit host specializations

(A,B) Aggression (A) and courtship (B) in *D. melanogaster*, *D. sechellia*, *D. santomea* (orange), and *D. erecta* male-male pairs competing over the standard apple juice substrate and other fruits based on reported specializations (*54*). Significance for each fruit/species combination by pairwise Mann-Whitney *U* tests to apple. Removing the food source significantly reduces aggression in *D. melanogaster* (*51*). *Morinda* (also called noni) significantly increases aggression but not courtship among *D. sechellia* males. *D. santomea* aggression and courtship depend only weakly if at all on food presence and source. *Morinda* and fig juices obtained commercially, *Marula* (*Sclerocarya birrea*) and *Pandanus* (*Pandanus furcatus*) obtained from botanical gardens (table S1).

bioRxiv preprint doi: https://doi.org/10.1101/2025.10.14.682417; this version posted November 14, 2025. The copyright holder for this preprint (which was not certified by peer review) is the author/funder, who has granted bioRxiv a license to display the preprint in perpetuity. It is made available under aCC-BY 4.0 International license.

Figure S4. Male- and female-directed social behaviors by *D. melanogaster*, *D. simulans*, *D. santomea,* and *D. yakuba* males

(A,B) Ethograms representing male-male (A) and male-female (B) social behaviors for *melanogaster* subgroup species of interest. Phylogeny also includes *D. mauritiana* and *D. sechellia* from the *simulans* clade, *D. teissieri* from the *yakuba* clade, and *D. erecta* without associated ethograms. Female-directed ethograms represent expert annotation for three recordings of male-female pairs per species, with male flies similarly prepared by single-housing and interactions taking place under identical conditions. Male-directed ethograms reproduced from Fig. 1A for visual comparison to female-directed counterparts. Note nearly exclusive male-directed aggressive actions (blue nodes) by *D. melanogaster* and *D. simulans* males but prevalence of male-directed courtship actions (red nodes) by *D. santomea* and *D. yakuba* (though in *D. yakuba* total male-direcetd actions are many fewer on average than *D. santomea)*, matching the actions taken when paired with females.

bioRxiv preprint doi: https://doi.org/10.1101/2025.10.14.682417; this version posted November 14, 2025. The copyright holder for this preprint (which was not certified by peer review) is the author/funder, who has granted bioRxiv a license to display the preprint in perpetuity. It is made available under aCC-BY 4.0 International license.

Figure S5. Acoustic features of female- and male-directed courtship song by *D. santomea* males

(A,B) Representative courtship song traces by *D. santomea* males toward a female (A, pink) or male conspecific target fly (B, purple). Key acoustic features are indicated including pulse, pulse train, inter-pulse interval (IPI), clack, clack train, and inter-clack interval (ICI) (*63*). Traces reproduced and enlarged from Fig. 1I,J.

(C,D) Video stills of song recording chambers (*60*) containing *D. santomea* males courting either a female (C) or male (D). Isolated waveforms of single clacks and pulse trios shown below, with asterisks indicating event peaks. Scale bars, 1 mm.

(E-J) Quantitative acoustic analyses of female- (pink) and male-directed *D. santomea* courtship songs (purple). Clacks (E-G) and pulses (H-J) were identified by expert annotation from three recordings with each sex (clack, 32-103 events per recording; pulse, 140-346 events per recording). Data shown as “hemiviolin” plots in which female- and male-directed distributions are vertically mirrored. Distribution means are semicircles on the appropriate side. Note general similarity between female- and male-directed song features (significant differences based on Mann-Whitney *U* tests despite small difference magnitudes are often because so many data points make up the underlying distributions). Analyses of absolute event abundances were omitted since annotations captured only a fraction of events in each recording.

bioRxiv preprint doi: https://doi.org/10.1101/2025.10.14.682417; this version posted November 14, 2025. The copyright holder for this preprint (which was not certified by peer review) is the author/funder, who has granted bioRxiv a license to display the preprint in perpetuity. It is made available under aCC-BY 4.0 International license.

Figure S6. GC-MS profiling of *D. santomea* male and female cuticular hydrocarbons (CHCs)

(A,B) Absolute (A) and relative abundances (B) of 41 compounds identifiable as peaks in *D. santomea* male (purple) and female hexane extracts (pink). Individual measurements are made on ten pooled, age-matched adult flies (3 male, 2 female replicates). Calculation of absolute abundance uses known concentration of the internal standard (octadecane/C18, not shown) and normalizes for number of flies included in the extract. Relative abundances normalize to the summed absolute abundance of all compounds excluding the standard. Male-specific (Z)-11-octadecenyl acetate (cVA, purple) and monomorphic (Z)-7-tricosene (7-T, black) are indicated. Question marks in compound names indicate uncertainty in chemical assignment. See table S5 for retention times and diagnostic ions used to identify and assign CHCs.

bioRxiv preprint doi: https://doi.org/10.1101/2025.10.14.682417; this version posted November 14, 2025. The copyright holder for this preprint (which was not certified by peer review) is the author/funder, who has granted bioRxiv a license to display the preprint in perpetuity. It is made available under aCC-BY 4.0 International license.

Figure S7. 7-tricosene (7-T) abundance in males and females of various *Drosophila* species and additional 7-T add-back experiments

(A) Absolute 7-T abundances on males (purple) and females (pink) *Drosophila* measured by GC-MS on hexane extracts from ten pooled, age-matched adult flies (2-8 replicates each). Monomorphic species (*D. santomea, D. yakuba*, *D. simulans*) show similar 7-T abundance between sexes while dimorphic species show large differences (*D. erecta*, *D. melanogaster*). nd, not detected in either sex (*D. serrata*, *D. ananassae*, *D. willistoni*).

(B-D) Fraction of time *D. santomea* males spend courting conspecific (B) or heterospecific dead females (C,D) perfumed with 10% 7-T (20 µg/fly) or solvent control (hexane). Heterospecific female targets selected for having either low (*D. erecta*) or undetectable (*D. ana*) endogenous 7-T levels (from GC-MS, A). *D. santomea* female targets as positive controls. 7-T addition elicits courtship from *D. santomea* males in both heterospecific cases. Significance by Dunn's tests.

bioRxiv preprint doi: https://doi.org/10.1101/2025.10.14.682417; this version posted November 14, 2025. The copyright holder for this preprint (which was not certified by peer review) is the author/funder, who has granted bioRxiv a license to display the preprint in perpetuity. It is made available under aCC-BY 4.0 International license.

Figure S8. Reduced cVA on *D. santomea* males compared to *D. yakuba* and *D. erecta*

(A-C) Mirrored gas chromatograms representing male (purple, top) and female (pink, bottom) cuticular hydrocarbon (CHC) profiles of *D. santomea* (A), *D. yakuba* (B), and *D. erecta* (C). Individual measurements made on hexane extracts from ten pooled, age-matched adult flies (2-3 replicates each). Peaks representing male-specific (Z)-11-octadecenyl acetate (cVA) indicated. *D. santomea* chromatograms reproduced from Fig. 2A for visual comparison.

(D,E) Absolute (D) and relative abundances (E) of cVA on *D. santomea* (orange), *D. yakuba* (green), and *D. erecta* males. Calculation of absolute abundance uses known concentration of the internal standard (C18) and normalizes for number of flies included in the extract. Relative abundances normalize to the summed absolute abundance of all compounds detected excluding the standard. *D. santomea* males show 63% and 75% reduced cVA comparing absolute abundance means or 64% and 65% reductions comparing relative abundance means to *D. yakuba* and *D. erecta*, respectively.

bioRxiv preprint doi: https://doi.org/10.1101/2025.10.14.682417; this version posted November 14, 2025. The copyright holder for this preprint (which was not certified by peer review) is the author/funder, who has granted bioRxiv a license to display the preprint in perpetuity. It is made available under aCC-BY 4.0 International license.

Figure S9. CRISPR-Cas9 HDR knock-in strategy used to generate *D. santomea Or67d*^{Gal4}

(A) Schematic diagrams of the Gal4 transgene and selection marker assembled into an Atalanta vector (pJAT32, addgene #204297) (*158*) and *Or67d* genomic target locus shown with upstream and downstream sgRNA cleavage sites. Transgene (above) contains two cassettes: *T2A-Gal4-stop(hsp70)* oriented natively to the genomic target, and downstream *ie1-mCherry-stop(p10)* fluorescent marker for successful integration (*32*) with inverted orientation. One kilobase homology arms flank both ends. *Or67d* genomic locus (below) is targeted using sgRNAs directing double-strand breaks to immediately (15 bp) downstream of the translational start codon and 348 bp downstream of the stop, resulting in removal of 1152 of 1167 protein coding base pairs (99%). Co-injection of the assembled Atalanta vector and nls-Cas9-nls protein into wildtype *D. santomea* eggs gives mosaic *ie1-mCherry*^{pos} G0 progeny carrying *T2A-Gal4* inserted in-frame with the five residual residues of *Or67d*. G1 progeny resulting from G0 crosses to wildtype are re-screened for abdominal *ie1-mCherry* expression to ensure germline transmission. In this strategy spatiotemporal patterns and magnitude of Gal4 expression are controlled entirely by *Or67d* endogenous genomic regulation.

(B,C) Photomicrographs showing labeling patterns of *Or67d*^{Gal4} in the whole brain of a *D. santomea* male (B, same fly from which close-up of antennal lobes (ALs) is shown in Fig. 3F) and female (C). Immunostaining for Gal4-dependent cytoplasmic tdTomato pseudocolored green with Bruchpilot synaptic counterstain (using nc82 monoclonal antibody) in magenta. Uniglomerular labeling pattern in each AL (arrowheads) matches the expected size and position of cVA-sensitive DA1 targeted by Or67d OSNs (*145*). Bifurcating OSN commissures (axon bundles) from the antennae leading into ALs can also be seen. Scale bars, 50 µm.

bioRxiv preprint doi: https://doi.org/10.1101/2025.10.14.682417; this version posted November 14, 2025. The copyright holder for this preprint (which was not certified by peer review) is the author/funder, who has granted bioRxiv a license to display the preprint in perpetuity. It is made available under aCC-BY 4.0 International license.

Figure S10. Ineffectiveness of naturally varying cVA levels on various species to promote aggression by *D. santomea* males

(A) Absolute cVA abundance on *Drosophila* males measured by GC-MS on hexane extracts from ten pooled, age-matched adult flies (2-8 replicates each). Calculation uses known concentration of the internal standard (C18) and normalizes for number of flies included in the extract. *D. santomea* (orange), *D. yakuba* (green), and *D. erecta* measurements reproduced from fig. S8 for visual comparison to additional species. Means indicated above and phylogeny below.

(B) Spontaneous aggression (lunges per minute) exhibited by *D. santomea* males toward conspecific (orange) or heterospecific males (*D. yakuba* green, all others black) in single-housed pairs during twenty-minute interactions. Statistical groupings by Dunn's tests shown above and phylogeny below. Note similarly low or even decreased attack levels toward males of all species.

(C) Correlation between cVA abundance measured by GC-MS and aggression elicited from *D. santomea* males. Dashed line, linear fit. Adjusted R^2, significance by F-test for fit, and Pearson correlation (r) indicated at bottom right. Note marginally significant correlation driven mostly by low attack rates toward distant species (*D. serrata*, *D. ananassae*, *D. willistoni*) with many other pheromone changes in addition to low cVA. Three of four species with higher cVA abundance than *D. santomea* elicit less attack than observed among *D. santomea* males (*D. simulans* is the exception but the slight increase is non-significant). Data derived from (A,B).

bioRxiv preprint doi: https://doi.org/10.1101/2025.10.14.682417; this version posted November 14, 2025. The copyright holder for this preprint (which was not certified by peer review) is the author/funder, who has granted bioRxiv a license to display the preprint in perpetuity. It is made available under aCC-BY 4.0 International license.

Figure S11. Social behavior changes by *melanogaster* subgroup males toward *D. santomea* males

(A) Relative fraction of time *D. melanogaster*, *D. simulans*, and *D. yakuba* (green) males court conspecific males (first distribution of each pair) vs. *D. santomea* males (second distribution, orange). All measurements within each species normalized to the conspecific median and $\log_{10}$ transformed. Males of all three *melanogaster* subgroup species show significantly increased courtship toward *D. santomea* males by Mann-Whitney *U* tests, including *D. melanogaster* where 7-T (abundant on *D. santomea* males) is male-specific and sexually aversive (*71–74*).

(B) Similar to (A) but showing paired relative distributions for aggression within and between males of the three subgroup species. Note decreases in male-directed aggression by all three toward *D. santomea* (significant for *D. melanogaster* and *D. yakuba* by Mann-Whitney *U* tests).

(C) Heatmap showing median fraction time spent courting by males in conspecific (diagonal) and heterospecific male-male pairs (off-diagonal) among the four subgroup species. Rows ("courters") are males whose courtship is measured (UWE). Columns ("courtees") are males targeted by courtship. Gray entry where data not collected. Data derived from (A) and additional pairings. Horizontal dashed arrows identify relevant comparisons between conspecific male- and *D. santomea* male-directed courtship for visual aid.

(D) Similar heatmap showing median aggression (lunge rate) by male "attackers" (rows) and "attackees" (columns). Data derived from (B) and additional pairings. Note consistently decreased aggression toward *D. santomea* males.

(E) Summarized changes in courtship (first row) and aggression rates (second row) by heterospecific males toward *D. santomea* compared to conspecific rates for each, derived from data in (A,B). Percent increases in first row (courtship) and percent decreases in second (aggression).

bioRxiv preprint doi: https://doi.org/10.1101/2025.10.14.682417; this version posted November 14, 2025. The copyright holder for this preprint (which was not certified by peer review) is the author/funder, who has granted bioRxiv a license to display the preprint in perpetuity. It is made available under aCC-BY 4.0 International license.

Figure S12. Induction of time-locked wing threat by photoactivation of AIP neurons in solitary *D. santomea* males

(A) Optogenetic photoactivation scheme for solitary *D. santomea* males carrying $20E08^{Gal4}$ and Gal4-dependent CsChrimson. Group-housed $20E08^{Gal4}$ tester males or genetic controls are exposed to two fifteen-second photostimulation (PS) blocks with a thirty-second baseline and inter-block interval.

(B) Manually scored wing threat rasters (purple) for solitary $20E08^{Gal4}$ males during the two-minute trial. Note threat induction time-locked to PS in most flies. Genetic controls showed no threat.

bioRxiv preprint doi: https://doi.org/10.1101/2025.10.14.682417; this version posted November 14, 2025. The copyright holder for this preprint (which was not certified by peer review) is the author/funder, who has granted bioRxiv a license to display the preprint in perpetuity. It is made available under aCC-BY 4.0 International license.

Figure S13. Induction of time-locked courtship by photoactivation of P1^{dsx} neurons in solitary *D. santomea* males

(A) Optogenetic photoactivation scheme for solitary group-housed *D. santomea* males carrying *P1*^{dsx} split-Gal4 (*71G01*^{DBD}*;dsx*^{AD}) and Gal4-dependent CsChrimson. *P1*^{dsx} tester males or a genetic control are exposed to six thirty-second photostimulation (PS) blocks with fixed intensity and monotonically increasing frequency as indicated, with a two-minute baseline and one-minute inter-block intervals.
(B) Courtship rasters (red) for *P1*^{dsx} tester males during the twelve-minute trial. Note early but sporadic courtship bouts that time-lock to PS blocks and increase in penetrance starting at 5 and 10 Hz.
(C,D) Penetrance (C) and expressivity (D) of courtship (UWE) elicited by *P1*^{dsx} PS. Significant courtship expression during PS blocks compared to the genetic control in (D) by Mann-Whitney *U* tests.

bioRxiv preprint doi: https://doi.org/10.1101/2025.10.14.682417; this version posted November 14, 2025. The copyright holder for this preprint (which was not certified by peer review) is the author/funder, who has granted bioRxiv a license to display the preprint in perpetuity. It is made available under aCC-BY 4.0 International license.

Figure S14. Induction of courtship and attack by photoactivation of $\mathbf{P1^{dsx}}$ neurons in same-genotype pairs of *D. santomea* males

(A) Optogenetic photoactivation scheme for same-genotype pairs of group-housed *D. santomea* males carrying $P1^{dsx}$ split-Gal4 and Gal4-dependent CsChrimson. $P1^{dsx}$ tester males or a genetic control are exposed to six thirty-second photostimulation (PS) blocks with fixed intensity and monotonically increasing frequency as indicated, with a two-minute baseline and one-minute inter-block intervals. **(B,C)** Behavior rasters for $P1^{dsx}$ male pairs (B) and genetic controls (C) during the twelve-minute trial. Blue, attack (lunge); red, courtship (UWE). Note early induction of attack which increases in penetrance and expressivity as the trial continues. Attacks interrupted by time-locked courtship during later PS blocks (>5 Hz). Genetic controls show little courtship or attack. **(D,E)** Locomotor dynamics in $P1^{dsx}$ males (browns) and genetic controls (black). Per-frame calculations of each fly's forward velocity (D) and angular velocity (E) derived from automated tracking (*56*). Data smoothed by averaging within a ten-second sliding window. Envelopes represent s.e.m. PS time-locked locomotor arrest similar to observed previously in *D. melanogaster* (*57*).

bioRxiv preprint doi: https://doi.org/10.1101/2025.10.14.682417; this version posted November 14, 2025. The copyright holder for this preprint (which was not certified by peer review) is the author/funder, who has granted bioRxiv a license to display the preprint in perpetuity. It is made available under aCC-BY 4.0 International license.

Figure S15. Photoactivation effects of *D. santomea* P1^{dsx} neurons in mixed-genotype male pairs

(A) Expressivity and temporal dynamics of attack (lunge) elicited by PS in mixed-genotype pairs between a *P1^{dsx}* split-Gal4 tester male carrying Gal4-dependent CsChrimson (or genetic control) and group-housed wildtype *D. santomea* target male. Traces show the mean fraction of frames containing lunge in a ten-second sliding window with s.e.m. envelopes for *P1^{dsx}* (blue) and control (black). PS block frequency, intensity, and timing indicated above for the twelve-minute trial. Note significant attack induction (Mann-Whitney *U* tests) in *P1^{dsx}* starting after 5 Hz.

(B) Attack elicited during inter-bout intervals and after final PS in same-genotype *P1^{dsx}* male pairs (blue, data from Fig. 4I) vs. mixed-genotype pairs between a *P1^{dsx}* tester and group-housed wildtype male (orange, from A). Period means connected with solid lines and data from individual flies shown as thin lines. Note significant right shift in mixed pairs (Mann-Whitney *U* tests) indicating higher PS frequencies required to induce intense attack.

(C) Action latencies for courtship and attack exhibited by *P1^{dsx}* tester males in same- and mixed-genotype pairs under identical PS trial structures. Data points derived from the same fly pair (or tester fly for mixed pairs) are connected as lines and distribution means shown as paired dots. Attack and courtship latencies are similar on average in same-genotype pairs whereas attack is significantly delayed in mixed pairs. Significance by paired or unpaired Mann-Whitney *U* tests (for within- or between-genotype group comparisons, respectively).

(D) Fraction of flies showing attack (lunge, blue) and courtship (UWE, red) in *P1^{dsx}* mixed-genotype pairs.

(E) Relative frequencies of *P1^{dsx}* same- and mixed-genotype pairs showing courtship (red) or attack (blue) first. Significance between distributions and between each distribution and a null probability of 50% (random chance) determined by binomial tests. Note courtship precedence in mixed pairs.

bioRxiv preprint doi: https://doi.org/10.1101/2025.10.14.682417; this version posted November 14, 2025. The copyright holder for this preprint (which was not certified by peer review) is the author/funder, who has granted bioRxiv a license to display the preprint in perpetuity. It is made available under aCC-BY 4.0 International license.

Figure S16. Unconditioned place preference tests with cVA in *D. melanogaster* and *D. santomea*

(A) Video still illustrating the y-maze arena. Four group-housed *D. melanogaster* males are introduced into the central bowl at the bottom and odors (10% cVA in acetone or acetone control) into ports at the head of each arm. Vacuum pressure continuously pulls air down the arms through an outlet. Flies can walk freely within the bowl and arms during ten-minute trials and odors are replaced into the same port (to avoid contamination) before each trial. Scale bar, 10 mm.

(B) Cumulative position trace representing movement of all four flies throughout the duration of the trial. Position density in each of multiple trials is used to calculate the odor preference index.

(C-H) Video stills and cumulative position traces as in (A,B) for *D. santomea* males (C,D), *D. melanogaster* females (E,F), and *D. santomea* females (G,H). Scale bars, 10 mm.

(I) Odor preference indices by species and sex. Index calculated as the difference in fly occupancy between the two arms normalized by the sum. Small gray dots represent single trials with 2-4 flies each (8-14 trials and 26-44 flies per species/sex). Distributions represented as violins with means shown as large black dots. Note significantly reduced attraction (or increased aversion) to 10% cVA in both *D. santomea* males and females (orange) compared to *D. melanogaster* (black). Significance by Mann-Whitney *U* tests.

bioRxiv preprint doi: https://doi.org/10.1101/2025.10.14.682417; this version posted November 14, 2025. The copyright holder for this preprint (which was not certified by peer review) is the author/funder, who has granted bioRxiv a license to display the preprint in perpetuity. It is made available under aCC-BY 4.0 International license.

Figure S17. cVA promotes *D. santomea* female sexual rejection during courtship by conspecific males

(A) Rasters of *D. santomea* female and male sexual behaviors during dyadic courtship interactions with increasing cVA on a nearby piece of filter paper. For each pair, male courtship (UWE, red) is shown above and female rejection (ovipositor extrusion, orange) or acceptance (wing spreading, cyan) below. Timing of copulations indicated with asterisks. cVA doses indicated at top. Note increased rejection and decreased copulation rates at 1 and 2 mg cVA.

bioRxiv preprint doi: https://doi.org/10.1101/2025.10.14.682417; this version posted November 14, 2025. The copyright holder for this preprint (which was not certified by peer review) is the author/funder, who has granted bioRxiv a license to display the preprint in perpetuity. It is made available under aCC-BY 4.0 International license.

Figure S18. Aggression and courtship biases in *melanogaster* subgroup male-male pairs

(A-D) Behavior rasters for three twenty-minute conspecific male-male pairs each of wildtype *D. melanogaster* (A), *D. simulans* (B), *D. yakuba* (C), and *D. santomea* (D) (same pairs as in Fig. 1A). Annotations of six aggressive interactions by manual scoring (blues) are shown above the horizontal dashed lines and five courtship actions (reds) beneath. Actions split by identity of the male to which they are attributed with the one exhibiting the greater total number of actions assigned as "Male 1." *D. melanogaster* and *D. simulans* males fight with some characteristic bias between flies, *D. yakuba* males are mostly passive but can either fight or court, and *D. santomea* males show frequent and elaborate courtship often with strong bias.

(E) Spontaneous action biases observed between flies in male-male pairs. Male-directed courtship (red dots) and attack (blue) are plotted as a function of their bout abundance (x-axis) and bias indices (y-axis). Measurements from the same pair connected by thin lines. Cases where the same fly is dominant for both actions show connections confined to the sector above the x-axis. Circled "X" indicates mean value for the corresponding action by color. Boxed regions indicate pairs with high attack expression and wide range of biases in *D. melanogaster* or high courtship expression and consistently strong bias in *D. santomea,* for visual aid.

bioRxiv preprint doi: https://doi.org/10.1101/2025.10.14.682417; this version posted November 14, 2025. The copyright holder for this preprint (which was not certified by peer review) is the author/funder, who has granted bioRxiv a license to display the preprint in perpetuity. It is made available under aCC-BY 4.0 International license.

Figure S19. Weak supporting evidence for territoriality in paired *D. santomea* males

(A) Video still of a pair of single-housed, wildtype *D. santomea* males in a large behavior chamber (40 mm wide x 50 mm long x 60 mm tall) containing an apple juice-based "food patch" (25 mm diameter) and neutral surround used to test territoriality. (A') Close-up of intermale courtship from boxed region. Scale bars, 10 mm (A), 1 mm (A').

(B-D) Heatmaps representing cumulative spatial patterns of all fly positions (B), attack (lunge) positions (C), and courtship (UWE) positions (D) for 17 *D. santomea* male-male pairs recorded during ten-minute interactions. Normalization in (B) to all frames, in (C) to attack frames only, and in (D) to courtship frames only. Note little sign of increased density on or around the food patch in any case, unlike previously observed for *D. melanogaster* aggression in similar assays (*51*).

(E) Quantification of (B-D) showing the fraction of time spent on the food patch in total, during attack, and during courtship for each individual *D. santomea* male. Normalizations as in heatmaps above. None are significantly different from having a null median of 25% (random chance, calculated as surface area of the food patch relative to the full arena) by one-sample Mann-Whitney *U* tests. Attack frames show near-significant deviation from chance, but in the opposite direction from that expected for territoriality (enrichment off the food patch).

(F) "Territoriality index" for *D. santomea* males calculated as the difference in time spent on the food patch between flies normalized by the sum. First distribution represents all pairs, second filtered for pairs where one of the flies showed attack dominance, and third filtered for pairs where one of the flies showed courtship dominance. None are significantly different from having a null median of zero (no territorial advantage) by one-sample Mann-Whitney *U* tests.

bioRxiv preprint doi: https://doi.org/10.1101/2025.10.14.682417; this version posted November 14, 2025. The copyright holder for this preprint (which was not certified by peer review) is the author/funder, who has granted bioRxiv a license to display the preprint in perpetuity. It is made available under aCC-BY 4.0 International license.

Figure S20. Additional behavior characterizations for the sliding door assay

(A) Summary of immediate and delayed behavioral outcomes in *D. santomea* males following brief $P1^{dsx}$ PS in the sliding door assay, with evidence below. $P1^{dsx}$ PS evokes immediate, time-locked courtship during the early isolation phase and in parallel generates a persistent internal state of social arousal. Subsequent encounters with conspecific females or males during the interaction phase elicit pure courtship or mixed courtship and aggression, respectively. Panels containing relevant data for each behavioral outcome are indicated. Figure modified from (*100*).

(B) Optogenetic photoactivation scheme for isolated *D. santomea* males carrying $P1^{dsx}$ split-Gal4 ($71G01^{DBD};dsx^{AD}$) and Gal4-dependent CsChrimson during the first phase of the delayed encounter ("sliding door") assay. After a one-minute baseline, two group-housed $P1^{dsx}$ tester males on either side of a removable divider are exposed to a one-minute photostimulation (PS) block. PS is followed by a ten-minute delay before doors are opened and flies allowed to interact. Delay phase shown as split into one- and nine-minute subphases to reflect cessation of detailed courtship annotations one minute after PS offset.

(C) Manually scored courtship rasters (UWE, red) for isolated $P1^{dsx}$ tester males during the first three minutes of the trial.

(D,E) Expressivity (C) and penetrance (D) of courtship elicited by PS. (C) Traces show the mean fraction of frames containing UWE in a ten-second sliding window with s.e.m. envelopes for $P1^{dsx}$ (red) and a genetic control (black, no courtship observed). (D) Fraction of flies showing courtship. Note extremely high penetrance and expressivity of induced courtship time-locked to PS in $P1^{dsx}$ testers.

(F,G) Fraction of $P1^{dsx}$ tester males showing courtship (red) and attack (blue) toward wildtype conspecific females (E) or males (F) during the ten-minute interaction phase after doors open. Exclusive courtship is observed toward females and a mix of courtship and attack toward males.

bioRxiv preprint doi: https://doi.org/10.1101/2025.10.14.682417; this version posted November 14, 2025. The copyright holder for this preprint (which was not certified by peer review) is the author/funder, who has granted bioRxiv a license to display the preprint in perpetuity. It is made available under aCC-BY 4.0 International license.

Figure S21. Aggression and courtship by *D. persimilis* and *D. pseudoobscura* males

(A,B) Behavior rasters for three twenty-minute conspecific male-male pairs each of wildtype *D. persimilis* (A) and *D. pseudoobscura* (B). Annotations of seven aggressive interactions by manual scoring (blues and black) are shown above the horizontal dashed lines and five courtship actions (reds) beneath. Actions split by identity of the male to which they are attributed with the one exhibiting the greater total number of actions assigned as “Male 1.” *D. persimilis* males court frequently including making copulation attempts (e.g., Pair 3). *D. pseudoobscura* also show some courtship but attack more intensely and often.

(C,D) Ethograms representing male- (C) and female-directed social behaviors (D) by *D. persimilis* and *D. pseudoobscura* males. Male-female ethograms represent expert annotation for three recordings of male-female pairs per species, with male flies similarly prepared by single-housing and interactions taking place under identical conditions. Male-directed ethograms reproduced from fig. S1A for visual comparison to female-directed counterparts. Note similarity between interactions with males and females in *D. per*.

(E) Spontaneous bias observed between flies in male-male *D. persimilis* and *D. pseudoobscura* pairs for attack and courtship. Indices derived from the same fly pair are connected as lines and distribution means shown as paired purple (*D. per*) or blue dots (*D. pse*). Both species show strong behavioral biases.

bioRxiv preprint doi: https://doi.org/10.1101/2025.10.14.682417; this version posted November 14, 2025. The copyright holder for this preprint (which was not certified by peer review) is the author/funder, who has granted bioRxiv a license to display the preprint in perpetuity. It is made available under aCC-BY 4.0 International license.

Ouadah, et al. fig. S1

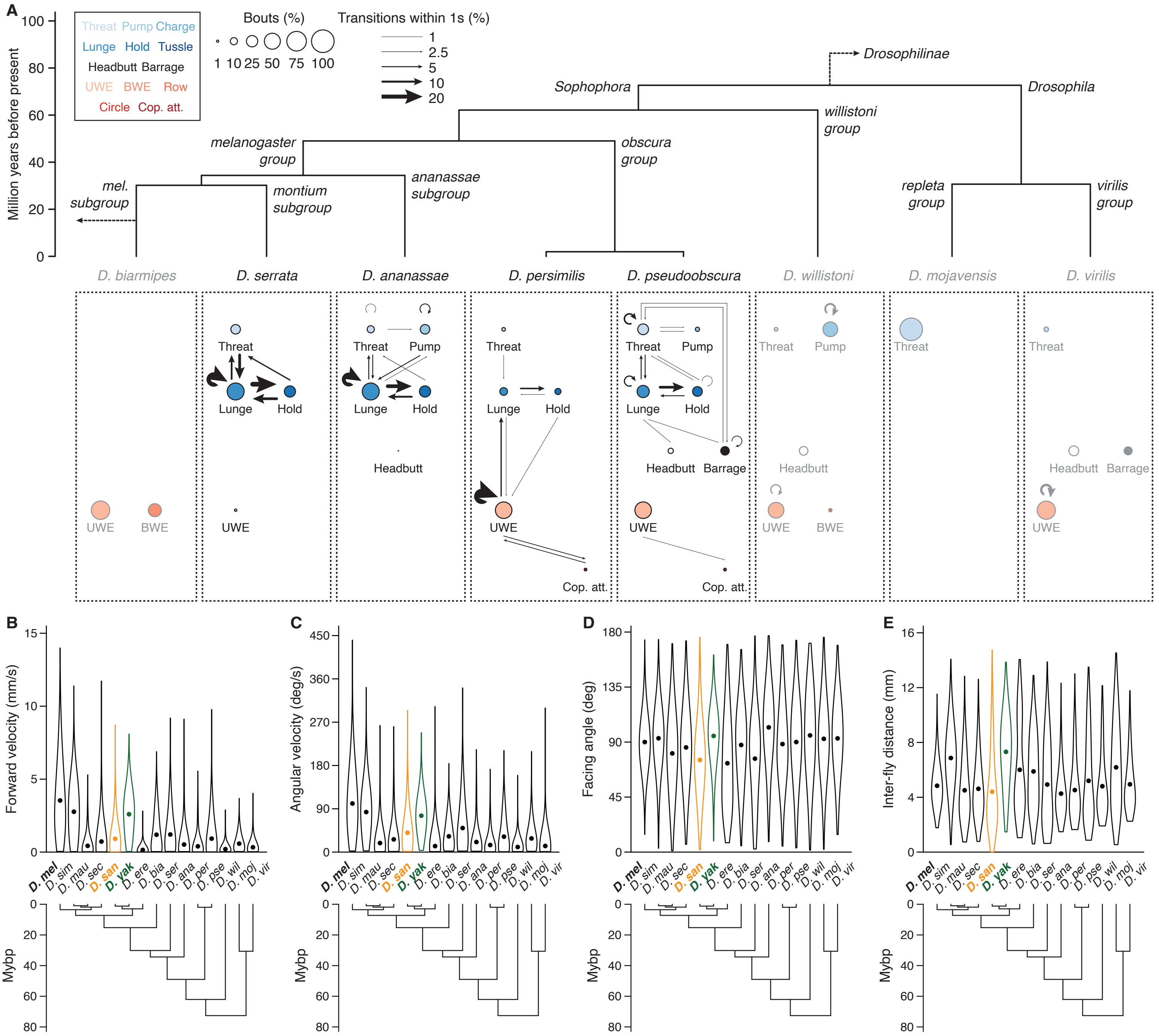

bioRxiv preprint doi: https://doi.org/10.1101/2025.10.14.682417; this version posted November 14, 2025. The copyright holder for this preprint (which was not certified by peer review) is the author/funder, who has granted bioRxiv a license to display the preprint in perpetuity. It is made available under aCC-BY 4.0 International license.

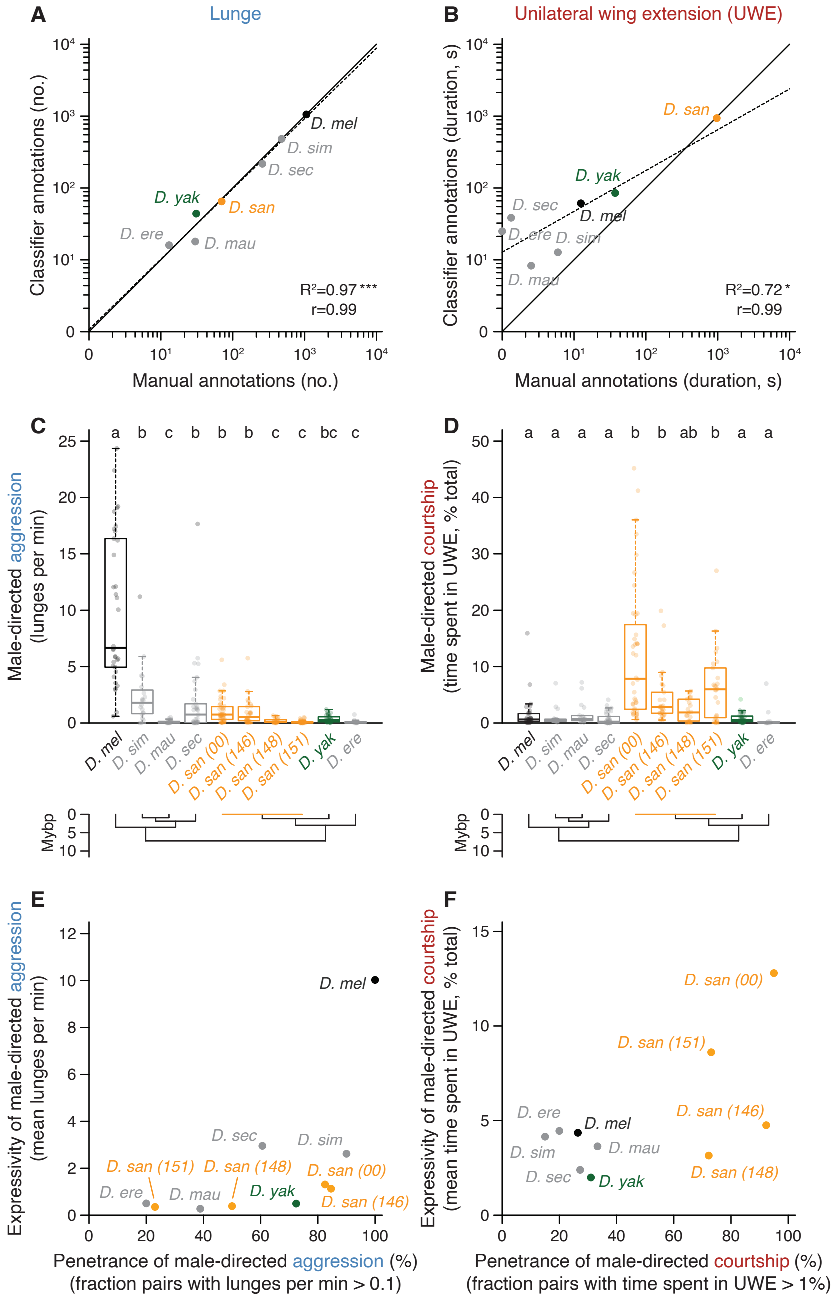

bioRxiv preprint doi: https://doi.org/10.1101/2025.10.14.682417; this version posted November 14, 2025. The copyright holder for this preprint (which was not certified by peer review) is the author/funder, who has granted bioRxiv a license to display the preprint in perpetuity. It is made available under aCC-BY 4.0 International license.

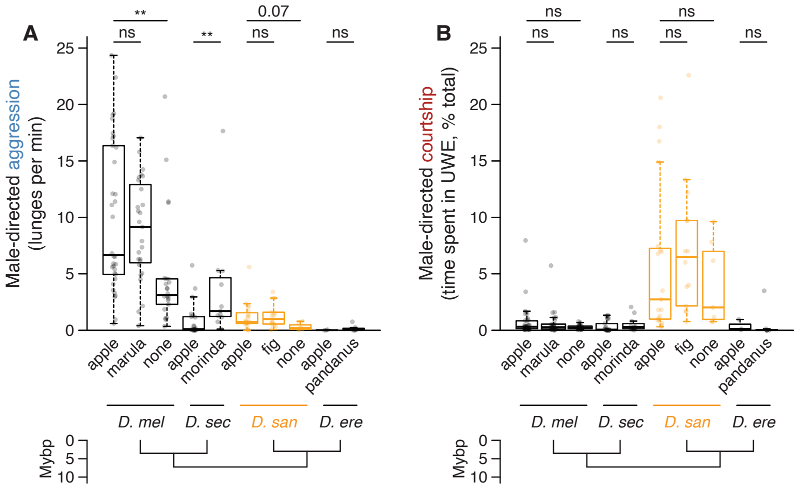

bioRxiv preprint doi: https://doi.org/10.1101/2025.10.14.682417; this version posted November 14, 2025. The copyright holder for this preprint (which was not certified by peer review) is the author/funder, who has granted bioRxiv a license to display the preprint in perpetuity. It is made available under aCC-BY 4.0 International license.

Ouadah, et al. fig. S4

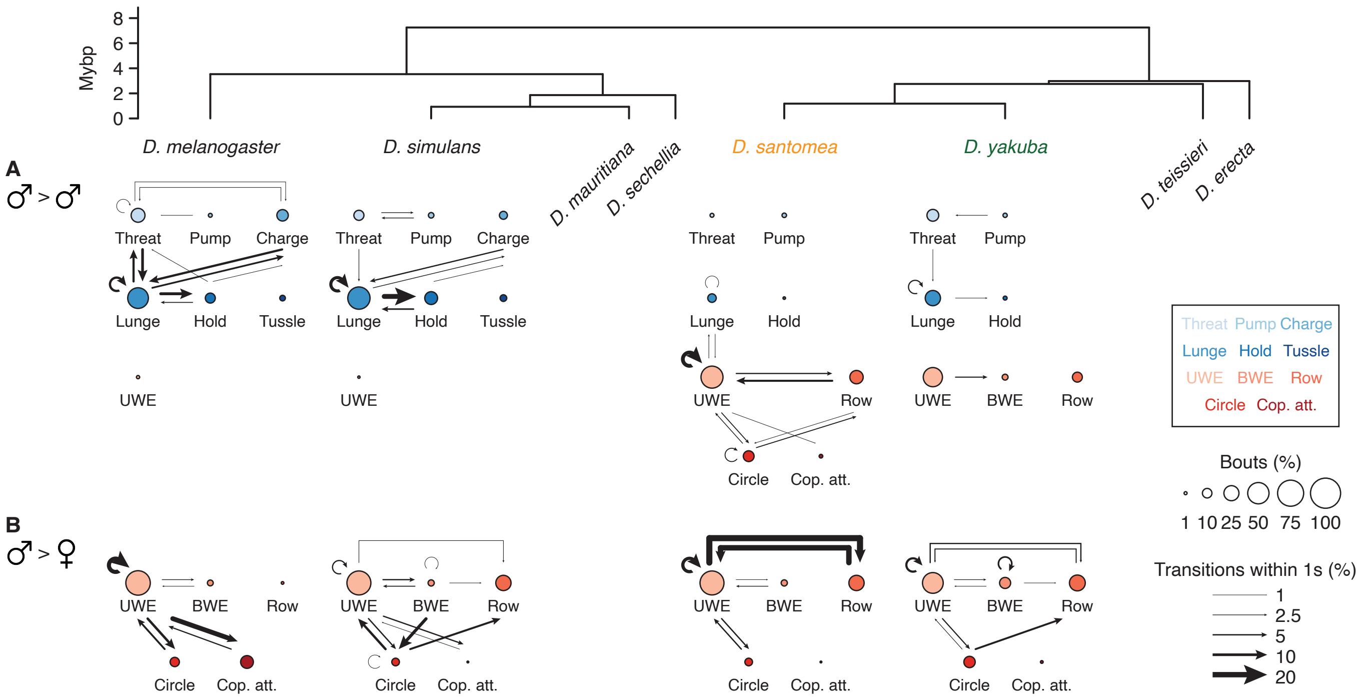

bioRxiv preprint doi: https://doi.org/10.1101/2025.10.14.682417; this version posted November 14, 2025. The copyright holder for this preprint (which was not certified by peer review) is the author/funder, who has granted bioRxiv a license to display the preprint in perpetuity. It is made available under aCC-BY 4.0 International license.

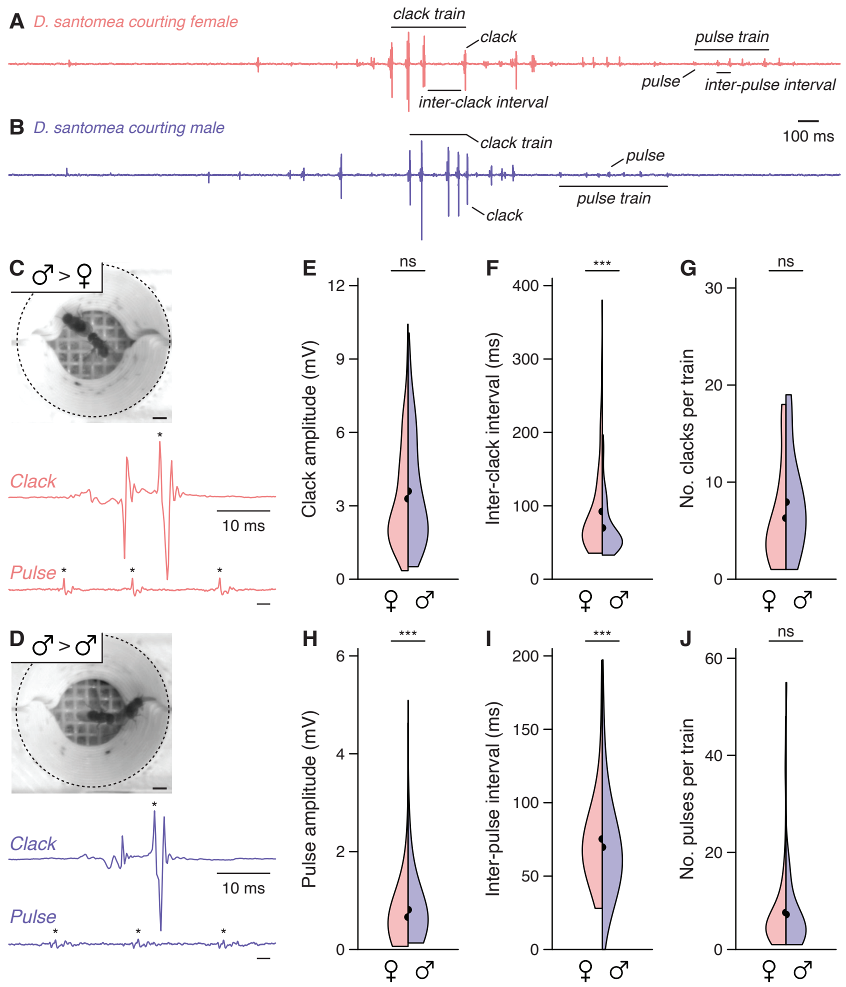

bioRxiv preprint doi: https://doi.org/10.1101/2025.10.14.682417; this version posted November 14, 2025. The copyright holder for this preprint (which was not certified by peer review) is the author/funder, who has granted bioRxiv a license to display the preprint in perpetuity. It is made available under aCC-BY 4.0 International license.

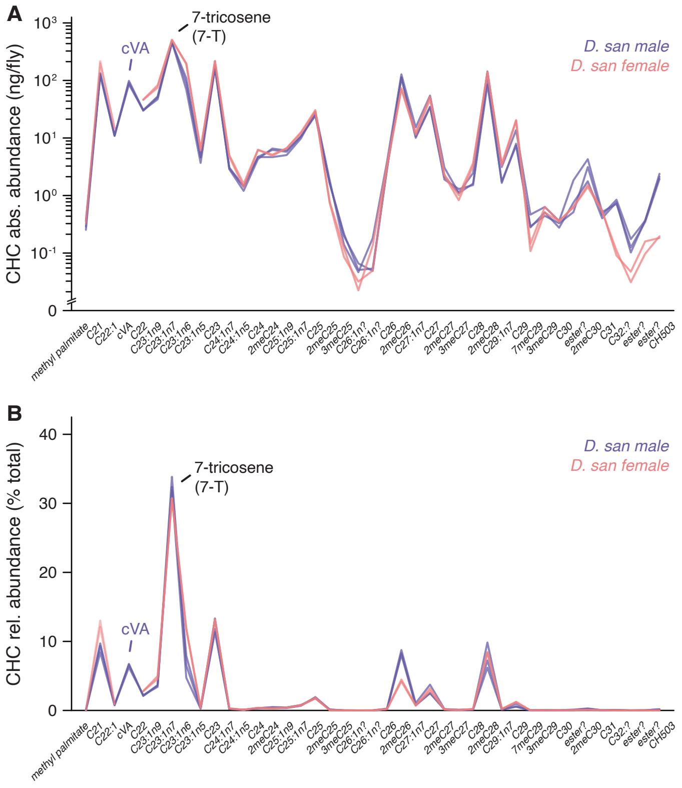

bioRxiv preprint doi: https://doi.org/10.1101/2025.10.14.682417; this version posted November 14, 2025. The copyright holder for this preprint (which was not certified by peer review) is the author/funder, who has granted bioRxiv a license to display the preprint in perpetuity. It is made available under aCC-BY 4.0 International license.

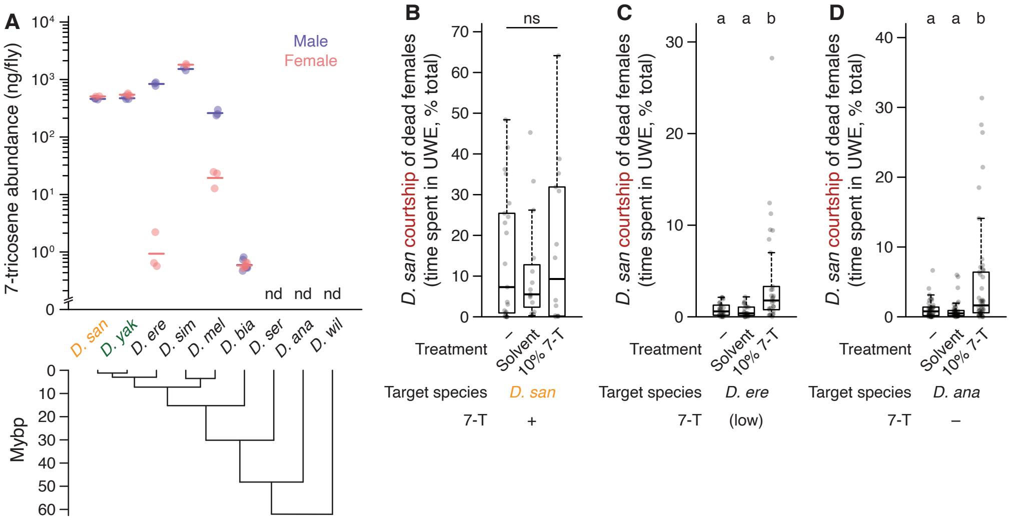

bioRxiv preprint doi: https://doi.org/10.1101/2025.10.14.682417; this version posted November 14, 2025. The copyright holder for this preprint (which was not certified by peer review) is the author/funder, who has granted bioRxiv a license to display the preprint in perpetuity. It is made available under aCC-BY 4.0 International license.

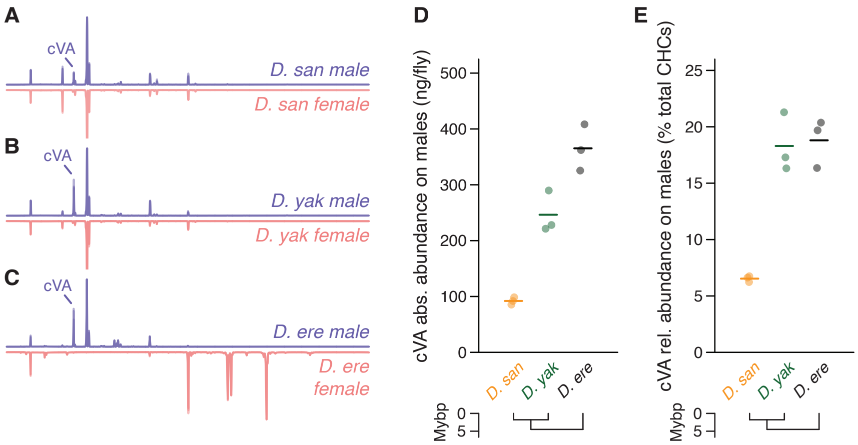

bioRxiv preprint doi: https://doi.org/10.1101/2025.10.14.682417; this version posted November 14, 2025. The copyright holder for this preprint (which was not certified by peer review) is the author/funder, who has granted bioRxiv a license to display the preprint in perpetuity. It is made available under aCC-BY 4.0 International license.

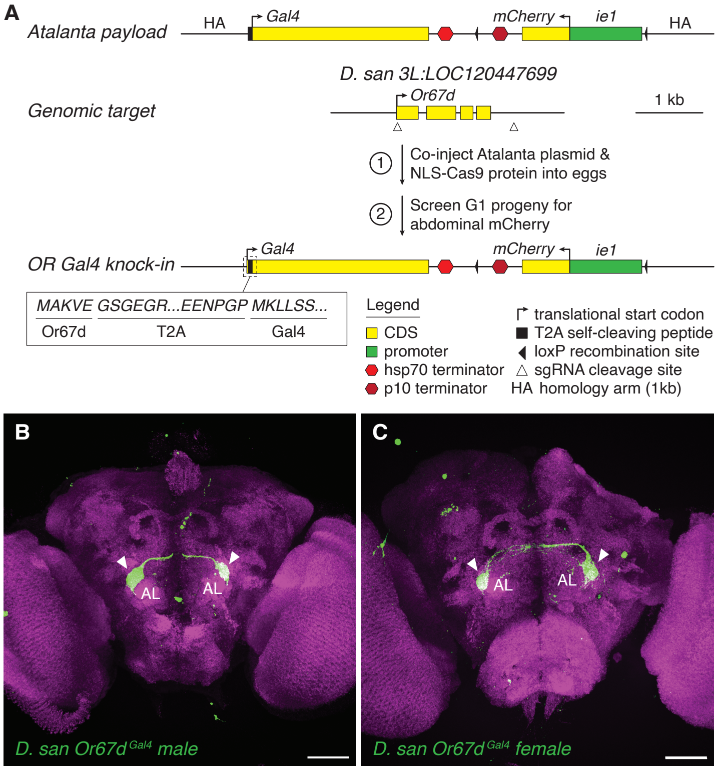

bioRxiv preprint doi: https://doi.org/10.1101/2025.10.14.682417; this version posted November 14, 2025. The copyright holder for this preprint (which was not certified by peer review) is the author/funder, who has granted bioRxiv a license to display the preprint in perpetuity. It is made available under aCC-BY 4.0 International license.

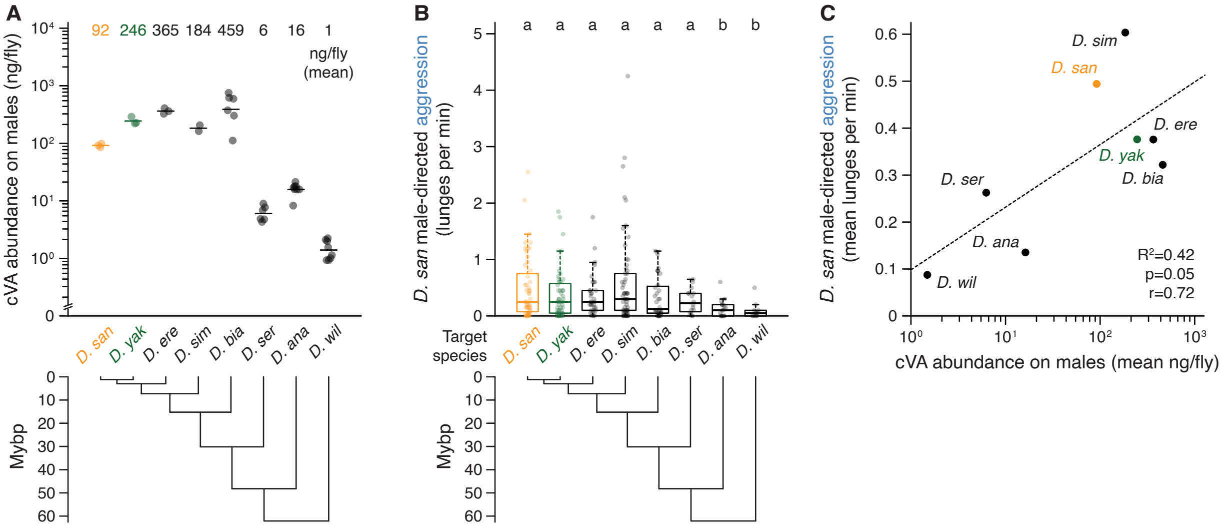

bioRxiv preprint doi: https://doi.org/10.1101/2025.10.14.682417; this version posted November 14, 2025. The copyright holder for this preprint (which was not certified by peer review) is the author/funder, who has granted bioRxiv a license to display the preprint in perpetuity. It is made available under aCC-BY 4.0 International license.

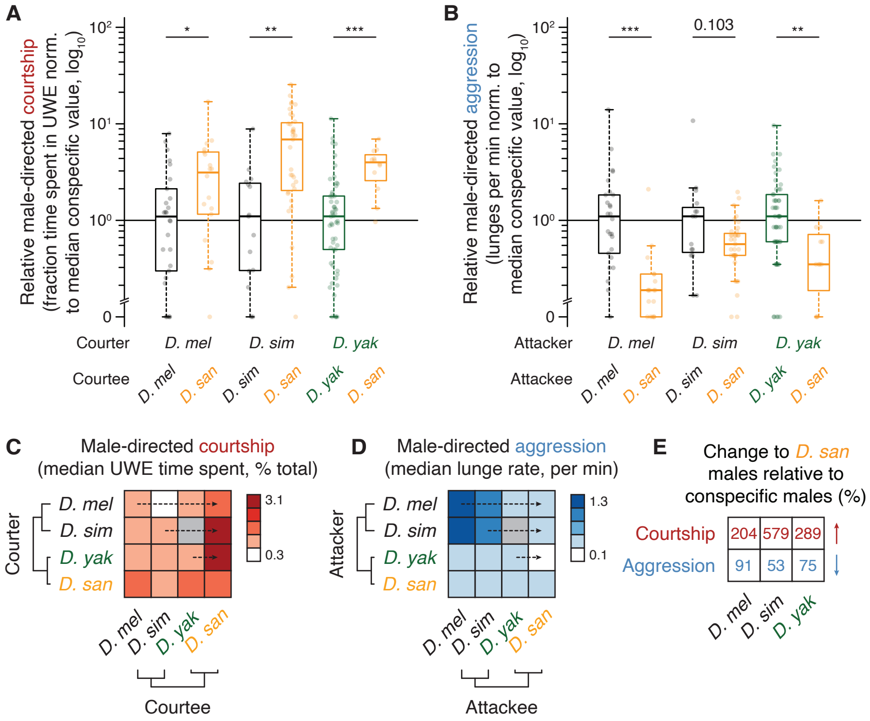

bioRxiv preprint doi: https://doi.org/10.1101/2025.10.14.682417; this version posted November 14, 2025. The copyright holder for this preprint (which was not certified by peer review) is the author/funder, who has granted bioRxiv a license to display the preprint in perpetuity. It is made available under aCC-BY 4.0 International license.

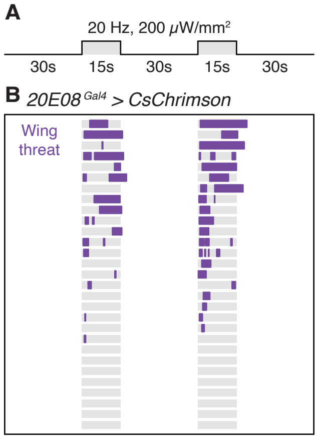

bioRxiv preprint doi: https://doi.org/10.1101/2025.10.14.682417; this version posted November 14, 2025. The copyright holder for this preprint (which was not certified by peer review) is the author/funder, who has granted bioRxiv a license to display the preprint in perpetuity. It is made available under aCC-BY 4.0 International license.

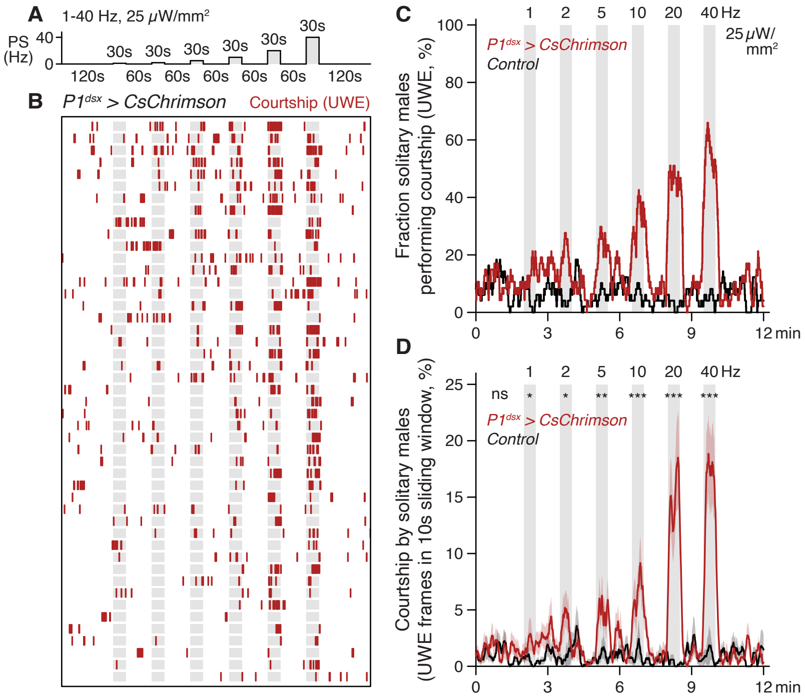

bioRxiv preprint doi: https://doi.org/10.1101/2025.10.14.682417; this version posted November 14, 2025. The copyright holder for this preprint (which was not certified by peer review) is the author/funder, who has granted bioRxiv a license to display the preprint in perpetuity. It is made available under aCC-BY 4.0 International license.

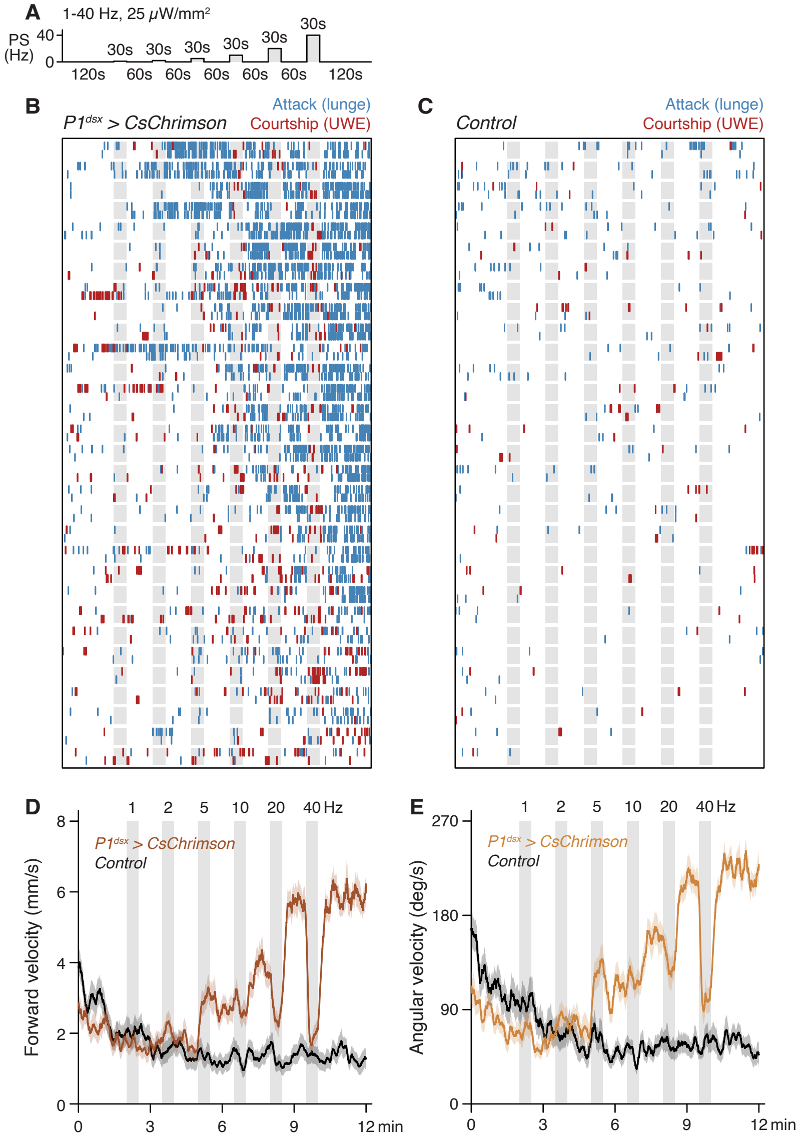

bioRxiv preprint doi: https://doi.org/10.1101/2025.10.14.682417; this version posted November 14, 2025. The copyright holder for this preprint (which was not certified by peer review) is the author/funder, who has granted bioRxiv a license to display the preprint in perpetuity. It is made available under aCC-BY 4.0 International license.

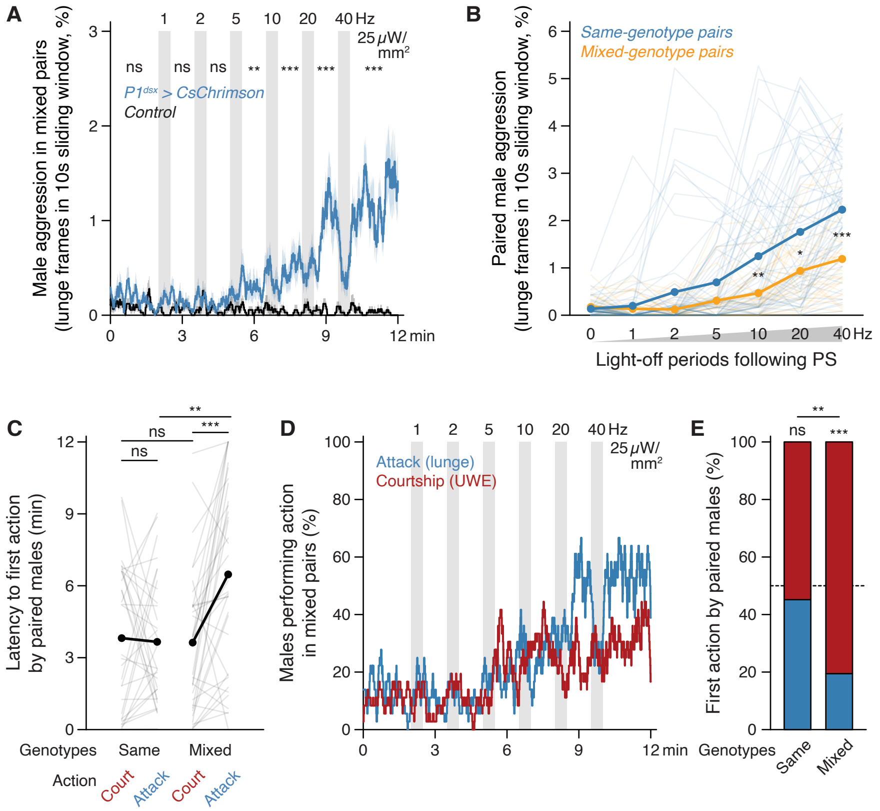

bioRxiv preprint doi: https://doi.org/10.1101/2025.10.14.682417; this version posted November 14, 2025. The copyright holder for this preprint (which was not certified by peer review) is the author/funder, who has granted bioRxiv a license to display the preprint in perpetuity. It is made available under aCC-BY 4.0 International license.

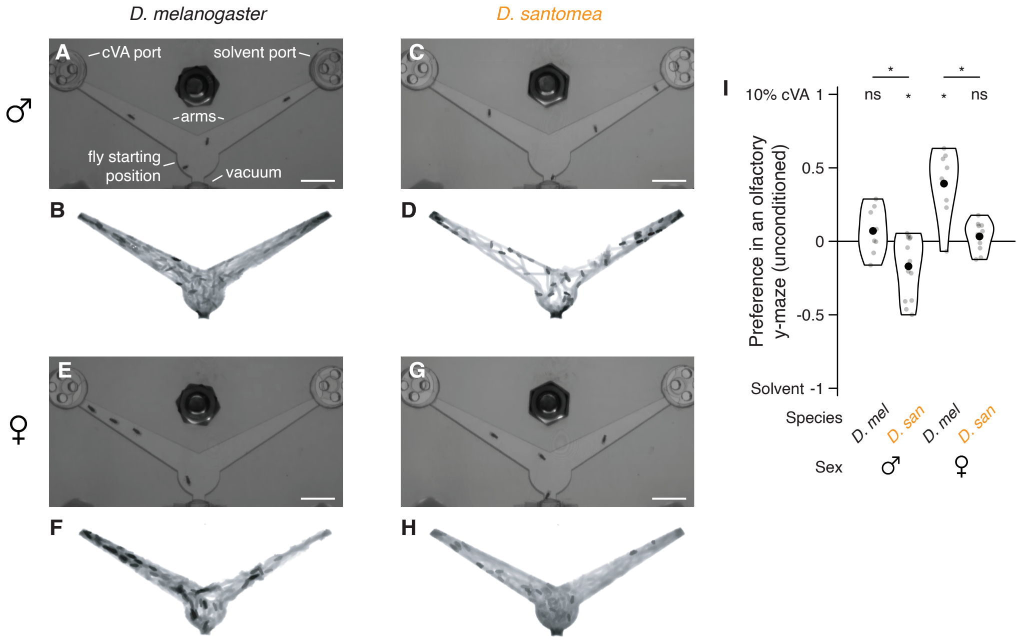

bioRxiv preprint doi: https://doi.org/10.1101/2025.10.14.682417; this version posted November 14, 2025. The copyright holder for this preprint (which was not certified by peer review) is the author/funder, who has granted bioRxiv a license to display the preprint in perpetuity. It is made available under aCC-BY 4.0 International license.

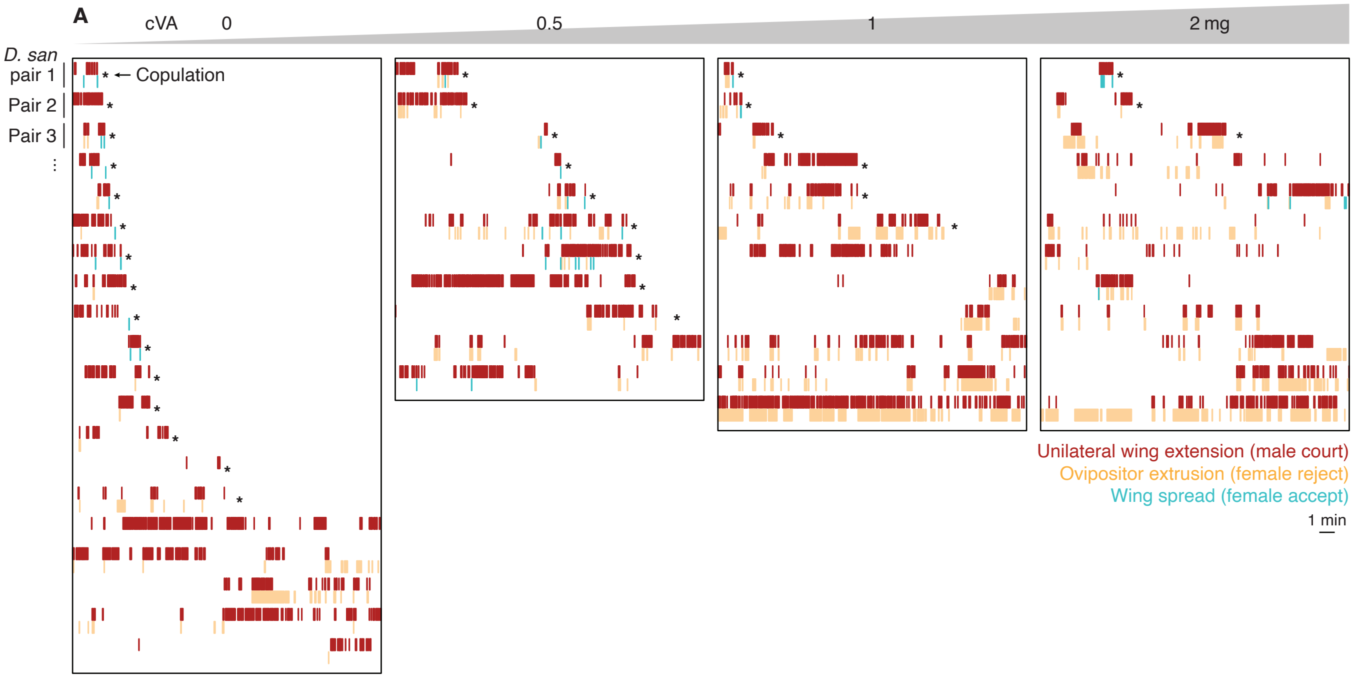

bioRxiv preprint doi: https://doi.org/10.1101/2025.10.14.682417; this version posted November 14, 2025. The copyright holder for this preprint (which was not certified by peer review) is the author/funder, who has granted bioRxiv a license to display the preprint in perpetuity. It is made available under aCC-BY 4.0 International license.

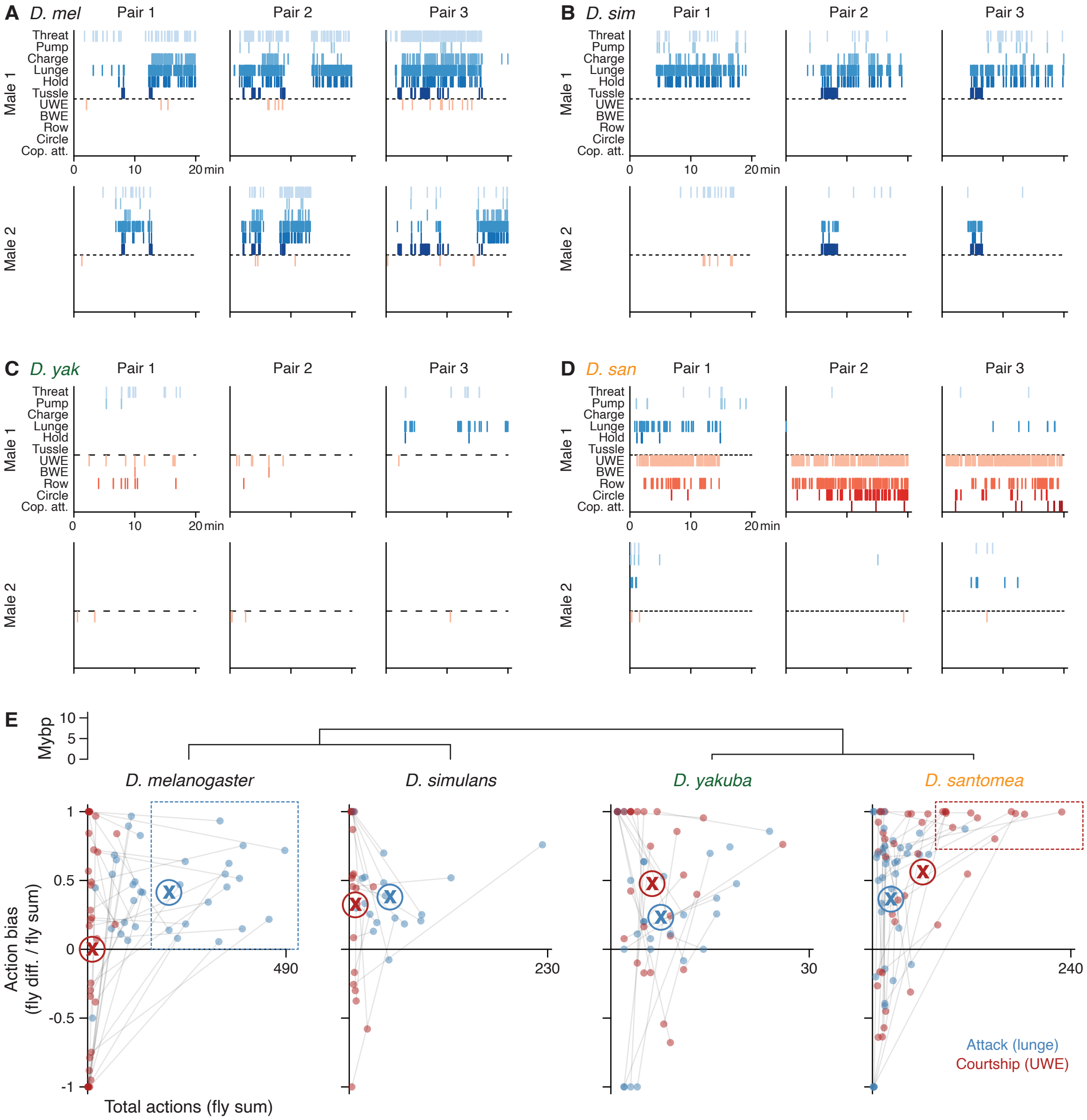

bioRxiv preprint doi: https://doi.org/10.1101/2025.10.14.682417; this version posted November 14, 2025. The copyright holder for this preprint (which was not certified by peer review) is the author/funder, who has granted bioRxiv a license to display the preprint in perpetuity. It is made available under aCC-BY 4.0 International license.

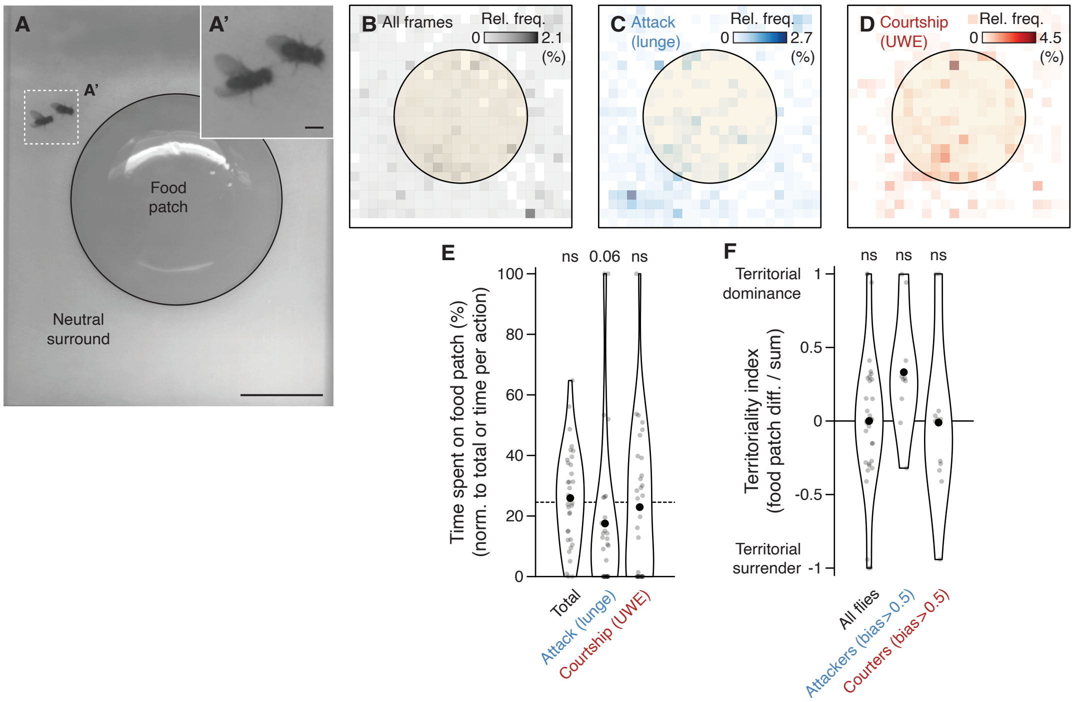

bioRxiv preprint doi: https://doi.org/10.1101/2025.10.14.682417; this version posted November 14, 2025. The copyright holder for this preprint (which was not certified by peer review) is the author/funder, who has granted bioRxiv a license to display the preprint in perpetuity. It is made available under aCC-BY 4.0 International license.

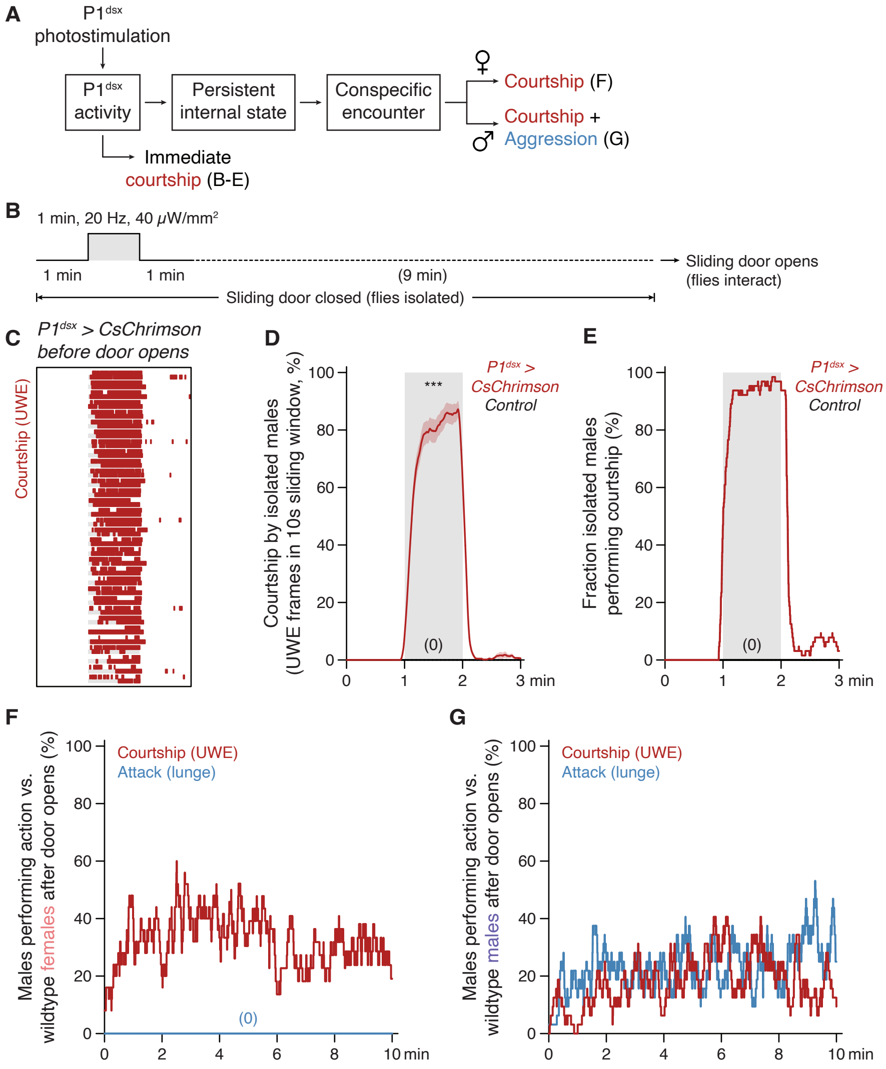

bioRxiv preprint doi: https://doi.org/10.1101/2025.10.14.682417; this version posted November 14, 2025. The copyright holder for this preprint (which was not certified by peer review) is the author/funder, who has granted bioRxiv a license to display the preprint in perpetuity. It is made available under aCC-BY 4.0 International license.

Ouadah, et al. fig. S21

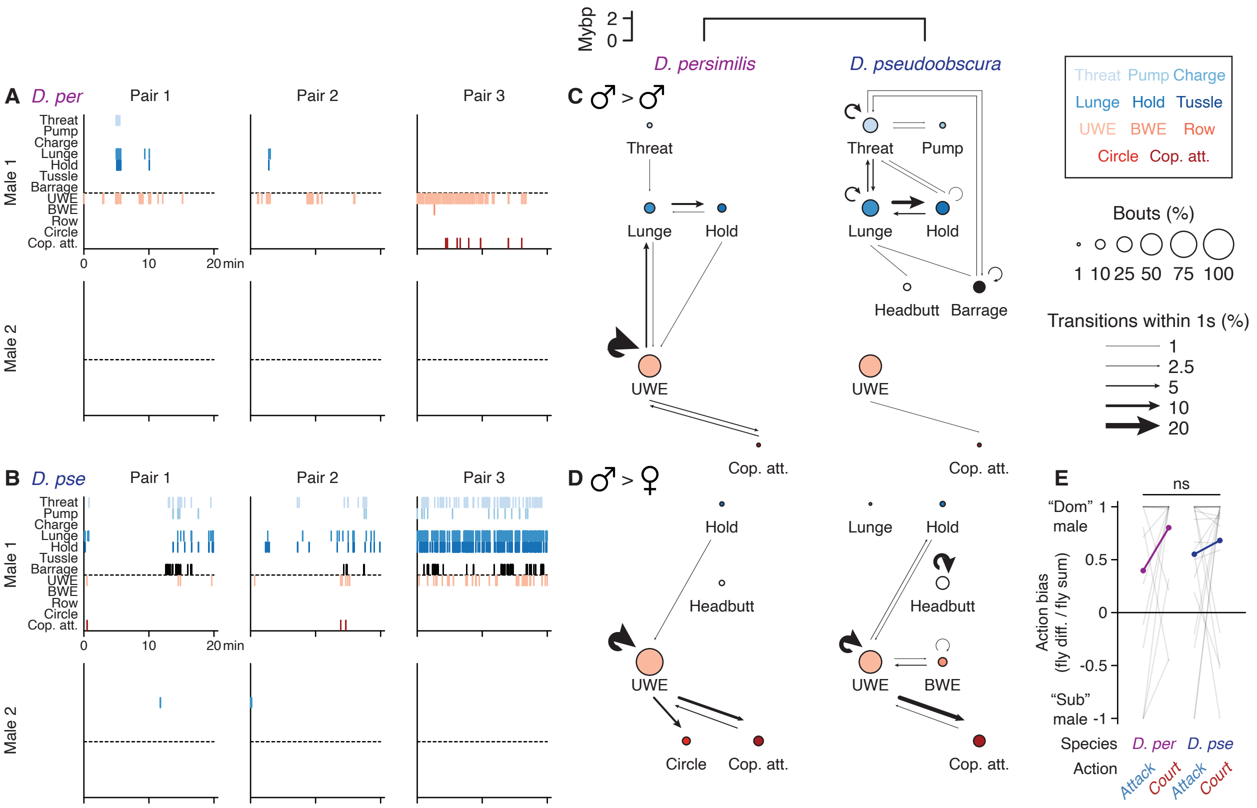

Supplement: 6 [file NIHPP2025.10.14.682417v2-supplement-6.pdf]
